# Comparison of Early and Mid-Term Outcomes After Classic and Modified Morrow Septal Myectomy in Patients with Hypertrophic Obstructive Cardiomyopathy

**DOI:** 10.21470/1678-9741-2023-0205

**Published:** 2024-01-30

**Authors:** Mustafa Karaarslan, Osman Fehmi Beyazal, Mehmed Yanartaş

**Affiliations:** 1 Department of Cardiovascular Surgery, Kartal Koşuyolu High Specialization Training and Research Hospital, İstanbul, Turkey; 2 Department of Cardiovascular Surgery, Başakşehir Çam and Sakura City Hospital, İstanbul, Turkey

**Keywords:** Cardiomyopathy Hypertrophic, Coronary Artery Bypass, Mitral Valve, Incidence, Heart Septum

## Abstract

**Introduction:**

The aim of our study is to compare the early and mid-term outcomes of patients with hypertrophic obstructive cardiomyopathy who underwent classic and modified Morrow septal myectomy.

**Methods:**

Between 2014 and 2019, 48 patients (24 males; mean age 49.27±16.41 years) who underwent septal myectomy were evaluated. The patients were divided into two groups - those who underwent classic septal myectomy (n=28) and those who underwent modified septal myectomy (n=20).

**Results:**

Mitral valve intervention was higher in the classic Morrow group than in the modified Morrow group, but there was no significant difference (P=0.42). Mortality was found to be lower in the modified Morrow group than in the classic Morrow group (P=0.01). In both groups, the mean immediate postoperative gradient was significantly higher than the mean of the 3^rd^ and 12^th^ postoperative months. The preoperative and postoperative gradient difference of the modified Morrow group was significantly higher than of the classic Morrow group (P<0.001).

**Conclusion:**

Classic Morrow and modified Morrow procedures are effective methods for reducing left ventricular outflow tract obstruction. The modified Morrow procedure was found to be superior to the classic Morrow procedure in terms of reducing the incidence of mitral valve intervention with the reduction of the left ventricular outflow tract gradient.

## INTRODUCTION

**Table:** 

Abbreviations, Acronyms & Symbols				
ASD	= Atrial septal defect		LVOT	= Left ventricular outflow tract
AV	= Atrioventricular		MR	= Mitral regurgitation
BMI	= Body mass index		MRA	= Mitral ring annuloplasty
CABG	= Coronary artery bypass grafting		MVR	= Mitral valve replacement
COPD	= Chronic obstructive pulmonary disease		NOAF	= New-onset atrial fibrillation
CPB	= Cardiopulmonary bypass		NSR	= Normal sinus rhythm
EF	= Ejection fraction		Q_1_	= First quarter
EuroSCORE	= European System for Cardiac Operative Risk Evaluation		Q_3_	= Third quarter
HOCM	= Hypertrophic obstructive cardiomyopathy		SAM	= Systolic anterior motion
ICU	= Intensive care unit		SD	= Standard deviation
IVS	= Interventricular septum		TTE	= Transthoracic echocardiographic
LAD	= Left atrial diameter		XCL	= Cross-clamping
LBBB	= Left bundle branch block			

Hypertrophic obstructive cardiomyopathy (HOCM) is a genetic disorder of the heart muscle characterized by a small left ventricular cavity, myocyte dysregulation, and marked hypertrophy of the myocardium^[1]^. Although medical treatment is the first-line treatment for symptomatic patients with left ventricular outflow tract (LVOT) obstruction, according to the 2020 American College of Cardiology/American Heart Association (or ACC/AHA) guidelines, in patients with HOCM who are severely symptomatic despite guided medical therapy, septal reduction therapy (SRT) in appropriate patients, performed in experienced centers, is recommended to relieve LVOT^[2]^.

The classic Morrow procedure was first described by Andrew Glenn Morrow in 1968^[3]^. In this classic procedure, a small muscle resection of the proximal interventricular septum (IVS) is performed to widen the LVOT, reducing systolic anterior motion (SAM) and relieving LVOT stenosis. Gao et al. described the modified Morrow procedure^[4]^. In this modified procedure, in addition to the classic Morrow procedure, the incision is extended to the midventricular region, widening the resection, and reducing the adhesions of papillary muscle and abnormal muscle bands combined with the IVS in the apical region. However, there are not many studies comparing these procedures.

In our study, we compared the early and mid-term results of patients with HOCM who underwent classic and modified Morrow septal myectomy.

## METHODS

This study was designed as a retrospective single-center study involving a total of 48 patients. The data of patients over the age of 18 years who underwent septal myectomy due to HOCM at the cardiovascular surgery clinic of Istanbul Kartal Koşuyolu High Specialization Training and Research Hospital between January 2014 and September 2019 were analyzed. Patients diagnosed with HOCM and symptomatic, with ventricular myocardial hypertrophy and a resting LVOT gradient > 35 mmHg, were included in the study. Patients with aortic valve stenosis, mitral valve stenosis, and discrete subaortic membrane diagnosis were excluded from the study. The patients were divided into two groups - those who underwent classic septal myectomy (n=28) and those who underwent modified septal myectomy (n=20).

All patients’ basic demographic information, medical history, laboratory parameters, surgical procedure details, preoperative and postoperative 3^rd^- and 12^th^-month transthoracic echocardiographic (TTE) data (IVS thickness, left atrial diameter [LAD], left ventricular posterior diameter, LVOT gradient, mitral regurgitation (MR) grade, and ventricular ejection fraction [EF]), and postoperative follow-up data were recorded by examining the hospital information management system.

Informed consent was obtained from the patients for this study. Ethics committee approval of Health Sciences University Kartal Koşuyolu High Specialization Training and Research Hospital was obtained for this study (2019.07.12).

### Statistics

In this study, biostatistical analyzes were performed with the NCSS - Number Cruncher Statistical System 2007 Statistical Software (Utah, United States of America) package program. In the evaluation of the data, besides descriptive statistical methods (mean, standard deviation, median, first quarter-third quarter), the distribution of variables was examined with the Shapiro-Wilk test of normality. The paired *t*-test was used for time comparisons of normally distributed variables, independent *t*-test was used for comparison of paired groups, Mann-Whitney U test was used for comparison of paired groups of variables that do not show normal distribution, chi-square test was used for comparisons of qualitative data, and Wilcoxon signed-rank test was used for time comparisons of qualitative data. The results were evaluated at the significance level of *P*<0.05.

## RESULTS

Demographic characteristics, clinical characteristics, intraoperative data, and postoperative data of the patients are shown in Table 1. The mean age of the patients was 51.79±16.02 years, and 24 (50%) were male. There was no statistical difference between the groups in terms of general demographic characteristics and comorbid conditions. Mitral valve replacement (MVR) was performed in nine (32.14%) patients in the classic Morrow group and in seven (35.0%) patients in the modified Morrow group, but no statistical difference was found. However, mitral ring annuloplasty (MRA) was performed in four (14.29%) patients in the classic Morrow group, while no MRA was performed in the modified Morrow group. Mitral valve intervention was higher in the classic Morrow group than in the modified Morrow group, but there was no statistically significant difference (13 [46.4%] - 7 [35%], respectively, *P*=0.42). There was no difference between the two groups in terms of coronary artery bypass grafting and atrial septal defect repair as a concomitant intervention. There was no statistical difference between the two groups in terms of cross-clamping (XCL) time, cardiopulmonary bypass time, and degree of hypothermia. There was no significant difference between the groups in terms of postoperative pacemaker need, new-onset atrial fibrillation (NOAF), total drainage amount, extubation time, intensive care unit (ICU) stay, and hospital stay. However, while seven (25%) patients in the classic Morrow group died, no mortality was observed in the modified Morrow group during the follow-up period, and it was found to be significantly lower in the modified Morrow group (*P*=0.01).

**Table 1:** Patients’ demographics, clinical characteristics, intraoperative data, and postoperative data.

	Classic Morrow (n=28)	Modified Morrow (n=20)	*P*-value
	Median (Q₁-Q₃), Mean±SD or n (%)	Median (Q₁-Q₃), Mean±SD or n (%)	
Sex, female	12 (42.86)	12 (60.0)	0.24^1^
Age (years)	51.79±16.02	45.75±16.7	0.21^2^
Height (cm)	163.29±8.06	160.75±9.57	0.32^2^
Weight (kg)	77.21±11.87	75±12.30	0.53^2^
BMI (m^2^)	29.09±5.15	29.01±3.95	0.95^2^
Diabetes mellitus	16 (57.14)	11 (55.0)	0.88^1^
COPD	12 (42.86)	5 (25.0)	0.20^1^
EuroSCORE II	0.8 (0.53-1.76)	0.8 (0.60-0.98)	0.65^3^
Intervention for mitral valve	13 (46.4)	7 (35.0)	0.42^1^
Concomitant MVR	9 (32.14)	7 (35.0)	0.83^1^
Concomitant MRA	4 (14.29)	0 (0)	0.13^4^
CABG	2 (7.14)	2 (10.0)	1.00^4^
ASD closure	1 (3.57)	1 (5.0)	1.00^4^
XCL time (min.)	76 (53.25-96.5)	73.5 (50.75-106.0)	0.73^3^
CPB time (min.)	111 (87.25-162)	118.0 (81.25-146.0)	0.86^3^
Hypothermia (°C)	28.64±2.41	29.45±2.37	0.25^3^
Internal pacemaker	2 (7.14)	1 (5.0)	0.76^1^
External pacemaker	9 (32.14)	4 (20.0)	0.35^1^
Atrial fibrillation	5 (17.86)	5 (25.0)	0.54^1^
Total drainage (ml)	450.0 (362.5-750.0)	500.0 (312.5-825.0)	0.90^3^
Extubation time (hours)	8.0 (6.0-12.0)	7.5 (6.0-12.0)	0.31^3^
ICU stay (days)	3.0 (2.0-4.0)	2.0 (1.0-4.75)	0.92^3^
Hospital stay (days)	7.5 (5.0-13.0)	7.5 (6.0-13.25)	0.64^3^
Mortality	7 (25.0)	0 (0)	0.01^1^

ASD=atrial septal defect; BMI=body mass index; CABG=coronary artery bypass grafting; COPD=chronic obstructive pulmonary disease; CPB=cardiopulmonary bypass; EuroSCORE=European System for Cardiac Operative Risk Evaluation; ICU=intensive care unit; MRA=mitral ring annuloplasty; MVR=mitral valve replacement; Q₁=first quarter; Q₃=third quarter; SD=standard deviation; XCL=cross-clamping

1 Chi-square test

2 Independent samples *t*-test

3 Mann-Whitney U test

4 Fisher’s exact test

The comparison of TTE findings of the classic Morrow and modified Morrow groups is shown in Table 2. There was no statistical difference between the two groups in terms of LVOT gradients in TTE findings at preoperative, immediate postoperative, and 3^rd^- and 12^th^-postoperative month TTE. In the classic Morrow group, the mean immediate postoperative gradient was significantly higher than the mean of the 3^rd^ and 12^th^ postoperative months (*P*=0.03, *P*=0.02). However, no significant difference was observed between the 3^rd^- and 12^th^-postoperative month gradients (*P*=0.15). In the modified Morrow group, the mean immediate postoperative gradient was significantly higher than the mean of the 3^rd^ and 12^th^ postoperative months (*P*=0.04, *P*=0.02). However, no significant difference was observed between the mean gradients of the 3^rd^ and 12^th^ postoperative months (*P*=0.76). There was no significant difference between the two groups in terms of preoperative IVS thickness, EF, posterior wall thickness, LAD, and moderate MR. No postoperative severe MR was observed in either group.

**Table 2:** Comparison of preoperative and postoperative echocardiographic findings between classic Morrow and modified Morrow groups.

	Classic Morrow (n=28)	Modified Morrow (n=20)	*P*-value
	Median (Q₁-Q₃), Mean±SD or n (%)	Median (Q₁-Q₃), Mean±SD or n (%)	
Preoperative gradient (mmHg)	75.0 (55.25-81.5)	77.5 (65.0-98.0)	0.22^1^
Postoperative gradient (mmHg)	37.0 (29.75-47.75)	31.5 (28.5-35.0)	0.11^1^
Gradient at postoperative 3 months (mmHg)	30.0 (23.75-33.5)	30.0 (21.0-31.0)	0.23^1^
Gradient at postoperative 12 months (mmHg)	28.0 (22.5-30.0)	28.0 (20.0-31.0)	0.81^1^
Preoperative IVS thickness (mm)	2.32±0.29	2.31±0.34	0.85^2^
Preoperative EF (%)	63.75±2.92	63.25±3.35	0.34^2^
Preoperative posterior wall (mm)	1.48±0.29	1.50±0.39	0.20^2^
Preoperative LAD (mm)	4.12±0.52	4.04±0.87	0.16^2^
Preoperative moderate MR	6 (21.43)	6 (30)	0.49^3^
Preoperative severe MR	9 (32.14)	4 (20)	0.35^3^
Postoperative IVS thickness (mm)	1.91±0.29	1.89±0.28	0.75^2^
Postoperative EF (%)	63.39±3.05	62.5±3.44	0.24^2^
Postoperative posterior wall (mm)	1.36±0.26	1.43±0.32	0.31^2^
Postoperative LAD (mm)	3.89±0.54	3.97±0.69	0.30^2^
Postoperative moderate MR	2 (7.14)	0 (0)	0.50^4^
Postoperative severe MR	0 (0)	0 (0)	-

EF=ejection fraction; IVS=interventricular septum; LAD=left atrial diameter; MR=mitral regurgitation; Q₁=first quarter; Q₃=third quarter; SD=standard deviation

1 Mann-Whitney U test

2 Student’s *t*-test

3 Chi-square test

4 Fisher’s exact test

The comparison of preoperative and postoperative electrocardiographic and TTE findings of the classic and modified Morrow groups is shown in Table 3. Postoperative IVS thickness, posterior wall thickness, and LAD of the classic Morrow group were found to be significantly lower than preoperative values (*P*<0.001, *P*=0.01, and *P*=0.01, respectively). In the modified Morrow group, postoperative IVS thickness and posterior wall thickness were found to be significantly lower than preoperative values (*P*<0.001 and *P*=0.03, respectively). However, there was no significant difference between LAD (*P*=0.72). There was no significant difference in terms of EF in the preoperative and postoperative periods in both groups.

**Table 3:** Comparison of preoperative and postoperative electrocardiographic and echocardiographic findings between classic and modified Morrow groups.

		Preoperative	Postoperative	*P*-value
		Median (Q1-Q3), Mean±SD or n (%)	Median (Q1-Q3), Mean±SD or n (%)	
Classic Morrow (n=28)	IVS thickness (mm)	2.32±0.29	1.91±0.29	< 0.001^1^
	EF (%)	63.75±2.92	63.39±3.05	0.41^1^
	Posterior wall (mm)	1.48±0.29	1.36±0.26	0.01^1^
	LAD (mm)	4.12±0.52	3.89±0.54	0.01^1^
	NSR	26 (92.86)	14 (50)	< 0.01^2^
	LBBB	2 (7.14)	8 (28.57)	0.03^2^
	AV block	0 (0)	6 (21.43)	0.01^2^
	Atrial fibrillation	0 (0)	5 (17.9)	0.02^2^
	Moderate MR	6 (21.43)	2 (7.14)	0.04^2^
	Severe MR	9 (32.14)	0 (0)	< 0.001^2^
Modified Morrow (n=20)	IVS thickness (mm)	2.31±0.34	1.89±0.28	< 0.001^1^
	EF (%)	63.25±3.35	62.5±3.44	0.08^1^
	Posterior wall (mm)	1.50±0.39	1.43±0.32	0.03^1^
	LAD (mm)	4.04±0.87	3.97±0.69	0.72^1^
	NSR	18 (90)	14 (70)	0.04^2^
	LBBB	2 (10)	5 (25)	0.08^2^
	AV block	0 (0)	1 (5)	0.02^2^
	Atrial fibrillation	0 (0)	5 (25)	0.02^2^
	Moderate MR	6 (30)	0 (0)	0.01^2^
	Severe MR	4 (20)	0 (0)	0.04^2^

AV=atrioventricular; EF=ejection fraction; IVS=interventricular septum; LAD=left atrial diameter; LBBB=left bundle branch block; MR=mitral regurgitation; NSR=normal sinus rhythm; Q₁=first quarter; Q₃=third quarter; SD=standard deviation

1 Independent Samples *t*-test

2 Wilcoxon signed-rank test

The comparison of the preoperative and postoperative gradient differences of the classic and modified Morrow groups is shown in Table 4. The preoperative and postoperative gradient difference of the modified Morrow group was significantly higher than of the classic Morrow group (∆70-∆39, *P*<0.001). There was no significant difference between the two groups in terms of preoperative and postoperative IVS thickness, EF, posterior wall thickness, and LAD differences. Although there was no significant difference between the two groups in terms of preoperative and postoperative MR, MR was worse in one patient in the classic Morrow group during the follow-up period.

**Table 4:** Comparison of preoperative and postoperative gradient differences between classic and modified Morrow groups.

		Classic Morrow (n=28)	Modified Morrow (n=20)	*P*-value
		Median (Q1-Q3) or n (%)	Median (Q1-Q3) or n (%)	
∆ Gradient		39.0 (26.0-50.0)	70.0 (61.25-81.5)	< 0.001^1^
∆ IVS thickness		0.40 (0.3-0.6)	0.45 (0.33-0.5)	0.95^1^
∆ EF		0 (0-0)	0 (0-0)	0.55^1^
∆ Posterior wall		0 (0-0.1)	0.05 (0-0.18)	0.47^1^
∆ LAD		0.20 (0-0.5)	0.25 (-0.43-0.4)	0.46^1^
∆ MR	Same	11 (39.29)	8 (40)	0.96^1^
	Better	16 (57.14)	12 (60)	0.84^1^
	Worse	1 (3.57)	0 (0)	0.39^1^

EF=ejection fraction; IVS=interventricular septum; LAD=left atrial diameter; MR=mitral regurgitation; Q₁=first Quarter; Q₃=third Quarter

1 Mann-Whitney U test

## DİSCUSSİON

HOCM is a disease characterized by diverse clinical features, including the risk of sudden death from arrhythmia, diastolic dysfunction, or LVOT obstruction, which is the major determinant of progressive heart failure^[5]^. Geometric changes in the LVOT, septal hypertrophy, and SAM of the mitral valve create varying degrees of obstruction in the LVOT, producing a gradient, and symptomatic HOCM develops^[6]^. Septal myectomy is a method that can be performed with low morbidity and mortality in patients who do not respond to medical treatment^[7]^. Although the classic Morrow procedure has been used for many years, many variations of this procedure have been reported^[8]^. Since there are not many studies in the literature to compare the results of these procedures, we designed this study.

According to the study by Lai et al. comparing classic and modified Morrow procedures, both the classic procedure and the modified procedure can reduce LVOT obstruction and relieve symptoms in patients with HOCM^[9]^. In addition, the modified Morrow septal myectomy was superior to the classic procedure in reducing the LVOT gradient with a lower incidence of MVR. According to the study of Song et al., modified Morrow septal myectomy is a safe and effective method of treating patients with HOCM and is superior to the conventional procedure in reducing the LVOT gradient and rate, restoring the normal anatomical atrioventricular size, and alleviating HOCM-related symptoms^[10]^. Similarly, in our study, the postoperative LVOT gradient decreased significantly compared to the preoperative LVOT gradient in both classic and modified Morrow procedures. In addition, the gradients at the 3^rd^ and 12^th^ postoperative months were also found to be significantly lower than the preoperative LVOT gradient and decreased over time. Although the preoperative and postoperative LVOT gradients were statistically similar for both procedures, the LVOT gradient of the modified Morrow group was higher than that of the classic Morrow group, while the postoperative gradients were lower than the preoperative gradient. The difference between preoperative and postoperative gradient reduction was significantly higher in the modified group than in the classic group. These results may indicate that the modified Morrow group is more effective than the classic group in reducing the LVOT gradient. In addition, IVS thickness, LAD, and posterior wall thickness decreased in the postoperative period compared to the preoperative period in both groups, and there was no significant difference between the groups. No severe MR was detected in the postoperative period in either group, and there was no significant difference between the groups, while the moderate MR decreased significantly. Accordingly, both procedures can be considered for the treatment of HOCM.

Mitral valve management is also important in HOCM surgery. Structural anomalies in the mitral valve, elongated leaflet, abnormally located papillary muscles, and chordae in the anterior leaflet can be seen in HOCM patients, and these anomalies may cause residual obstruction and SAM in the postoperative period if they are not managed properly during surgery^[11]^. When there are abnormal chordae tendineae adhered to the left ventricular free wall, IVS, or papillary muscle fusion intraoperatively, these should be removed^[12]^. Wider resection with the modified Morrow procedure may be a more effective solution to the problem of papillary muscle fusion, and patients may have better postoperative clinical outcomes^[13]^. In the study by Lai et al., no significant difference was found in the classic and modified Morrow groups in terms of mitral valvuloplasty, but the MVR rate was found to be lower in the modified group^[9]^. In our study, although the MVR rates were similar in both groups, interventions for the mitral valve were more frequent in the classic Morrow group than in the modified Morrow group, although it was not statistically significant. Since our patients in the modified group did not have MRA and only had MVR, there may not be a significant difference between the results. However, we think that this difference will be more pronounced in future studies with a higher number of patients because insufficient myectomy and some structural abnormalities in the mitral valve may cause a higher rate of intervention to the mitral valve in the classic group. In addition, MVR is important in terms of eliminating SAM symptoms.

There was no significant difference between the groups in terms of postoperative pacemaker need, NOAF, total drainage, extubation time, ICU stay, and hospital stay. However, it was observed that the mortality rate was higher in the classic Morrow group compared to the modified group, and these deaths were generally due to low cardiac output in the early postoperative period. In the classic Morrow group, although not statistically significant, the higher rate of intervention for the mitral valve, the rate of severe MR and comorbid conditions, the higher postoperative gradient, lower gradient change, longer XCL time, and longer ICU stay may cause this situation. However, these results need to be better clarified by future prospective studies with a larger number of patients. In conclusion, when we look at all these results, we think that the classic and modified Morrow procedures may be preferred in the treatment of HOCM to reduce LVOT obstruction, and the modified group is superior to the classic group by reducing the intervention rate for the mitral valve.

### Limitations

The most important limitations of this study are its retrospective, single-center design and the limited number of patients. In addition, due to the retrospective study design, we could not perform some analyses because we could not reach the TTE findings sufficiently. Since there were no long-term results, we could not evaluate some parameters such as the need for reintervention for the mitral valve.

## CONCLUSİON

Classic Morrow and modified Morrow procedures are effective methods for reducing LVOT obstruction. The modified Morrow procedure was found to be superior to the classic Morrow procedure in terms of reducing the incidence of mitral valve intervention with the reduction of the LVOT gradient. Despite the limited number of patients, the data obtained from this study will guide larger, prospective studies.

**Table:** 

Authors’ Roles & Responsibilities	
MK	Substantial contributions to the conception or design of the work; and the acquisition, analysis, and interpretation of data for the work; drafting the work or revising it critically for important intellectual content; final approval of the version to be published
OFB	Substantial contributions to the analysis of data for the work; drafting the work or revising it critically for important intellectual content; final approval of the version to be published
MY	Substantial contributions to the conception or design of the work; drafting the work or revising it critically for important intellectual content; final approval of the version to be published
